# Editorial: Streaming inflammation: From damage to healing and resilience–Volume II

**DOI:** 10.3389/fphar.2023.1185593

**Published:** 2023-03-24

**Authors:** Pallavi R. Devchand, Eric E. Schadt, Garret A. FitzGerald

**Affiliations:** ^1^ Department of Physiology and Pharmacology, University of Calgary, Calgary, AB, Canada; ^2^ Department of Genetics and Genomic Sciences, Icahn School of Medicine at Mount Sinai, New York, NY, United States; ^3^ Department of Systems Pharmacology and Translational Therapeutics, Perelman School of Medicine, University of Pennsylvania, Philadelphia, PA, United States

**Keywords:** resilience, disease states, drug target, healing, identity, lipid mediators

“It takes a very long time to become young.”

-*Pablo Ruiz Picasso* (1881-1973)

Age is a kaleidoscope of identity. It mirrors a number, a malady, a development, an insult and … even a compliment. In all dimensions of time, age reflects the fine balance between adaptability and integrity. Our first volume on Streaming Inflammation deemed every human as a multiplex of ecosystems (Devchand et al.). Here, we explore the impacts of damage, healing and resilience on the plasticity of identity as we age.

Longitudinal studies emphasize that identity is fluid. Sayah et al. demonstrate how optical coherence tomography imaging coupled with an automated segmentation algorithm can be applied to study dynamic cellular responses during eye development. This non-invasive method coupled with selective-receptor modulation during oxygen-induced retinopathy provides a powerful approach to understanding retinopathy of prematurity in small rodents. In humans, using real-world dynamics of the JIR cohort of patients with pediatric inflammatory diseases, Hentgen et al. tackle the dosing regiment of off-label use of Interleukin-1 inhibitors. Interestingly, in patients with a monogenic auto-inflammatory disease, the actual-doses used in treat-to-target data present an adaptive comparison with that of recommended drug dosage of the medications.

Although identity is personal, what we share in common also provides for targeted intervention in promoting healing. Hickey et al. take a computational approach to the chronic inflammation component of complex diseases. Innovatively applying *GenePlexus* supervised machine learning, they depict disease heterogeneity into gene clusters of disease-specific chronic inflammation. This facilitates imputing drug priority per disease cluster and identifying potential novel therapeutics. Meanwhile, Skaria et al. toggle Wnt-5A signaling to evaluate pharmacokinetics mediated by damage from innate immune responses on primary human coronary artery endothelial cells. Using transcriptomics and gene ontology analysis, they zone in on the potential need to reflect on the drug-metabolizing cytochrome P450 enzymes, specifically Cyp1A1/Cyp1B1.

Lipid mediators are potent instigators of change in identity. Yamaguchi et al. review the dynamics of bioactive lipids in the blood and vascular wall. The biosynthesis and mechanistic signaling are critically discussed in context of pro- and anti-inflammation. Bergqvist et al. developed a semi-high throughput bioassay measuring prostaglandin E_2_ production and IL-8 secretion from whole human blood. Interestingly, this study focus is on key epigenetic modulators and kinase inhibitors within the Structural Genomic Consortium, and aims to identify chemical probes that potentially trigger resolution of chronic inflammation.

Throughout life, the bone is a hub of remodeling of identity. Kalkar et al. focus on the interplay between immune cells and osteoclasts. They reveal an intriguing interplay between a nitrogen-containing bisphosphonate and a glucocorticoid that intersects at interferon-ß expression to inhibit osteoclastogenesis. This work has potential implications on prevention of osteolytic lesions post-chemotherapy.

Dietary intervention of immune activity is a universal story of age. In a didactic exercise, Wang et al. review the concept of trained immunity in atherosclerosis from perspective of metabolic reprogramming, epigenetic reprogramming and promotion of myelopoieisis progenitors. Substantive emphasis is placed on natural products that potentially have anti-atherosclerotic abilities *via* trained monocytes/macrophages. In a different tact, Zhou et al. focus their computational efforts on patients with rosacea. They zone in on an association between keratinocyte autophagy and the mammalian target of rapamycin (mTOR) pathway. After confirming this relationship in a mouse model, they use molecular docking analyses to pinpoint the natural polyphenol EGCG as a candidate lead for mTOR-pathway therapeutics.

Resilience often surfaces in the face of vulnerability. Cheng et al. perform a prospective study on the effective use of vercanozole in treatment of invasive fungal infections. Through stepwise multivariate linear regression analyses of young and elderly patients from a single-center cohort, they identify factors affecting drug trough and metabolite concentrations in plasma. Intriguing prospects for biomarkers are proposed for correlation to predictive effect of the drug.

**FIGURE 1:**
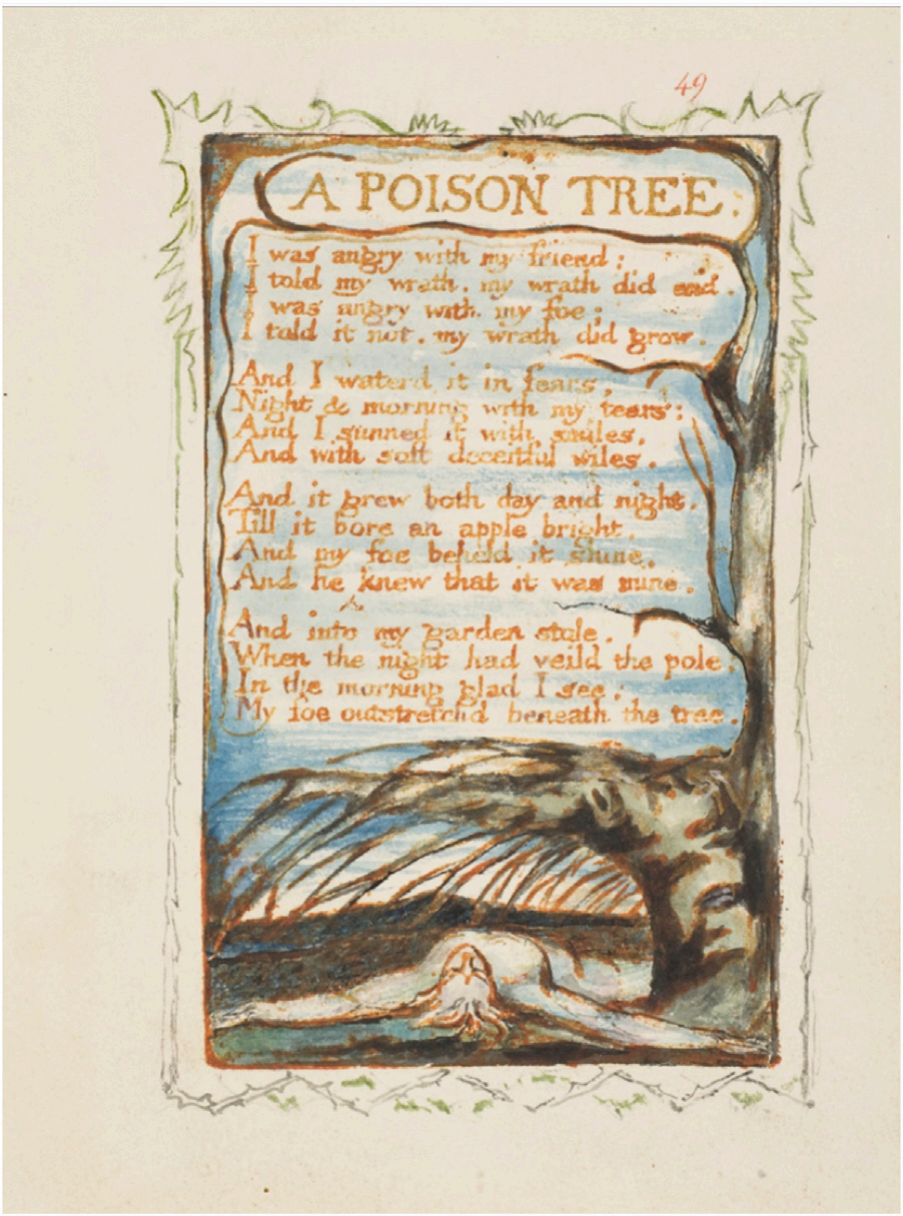
Songs of Experience: A Poison Tree. Plate 49. William Blake (British, London 1757-1827). The Metropolitan Museum of Art, New York, Rogers Fund, 1917. www.metmuseum.org. Image obtained under The Met’s Open Access progam.

Age is an art. Blake’s bright apple stems from damage, healing and resilience (Figure 1). Picasso’s Pomme is a small matter of fluid identity and relativity. And for larger-than-life Miriam Makeba (1932-2008),

“Age is getting to know all the ways the world turns, so that if you cannot turn the world the way you want, you can at least get out of the way, so you will not get run over.”

